# Gene methylation profile of gastric cancerous tissue according to tumor site in the stomach

**DOI:** 10.1186/s12885-016-2077-8

**Published:** 2016-01-26

**Authors:** Rita Kupcinskaite-Noreikiene, Rasa Ugenskiene, Alius Noreika, Viktoras Rudzianskas, Jurgita Gedminaite, Jurgita Skieceviciene, Elona Juozaityte

**Affiliations:** Oncology Institute of the Lithuanian University of Health Sciences, Eiveniu 2, Kaunas, 50009 Lithuania; Kaunas University of Technology, Studentu 50, Kaunas, 50009 Lithuania; Laboratory of Clinical and Molecular Gastroenterology, Lithuanian University of Health Sciences, Eiveniu 2, Kaunas, 50009 Lithuania

**Keywords:** Stomach cancer, *MLH1*, *DAPK-1*, *MGMT*, Methylation

## Abstract

**Background:**

There is considerable information on the methylation of the promoter regions of different genes involved in gastric carcinogenesis. However, there is a lack of information on how this epigenetic process differs in tumors originating at different sites in the stomach.

The aim of this study is to assess the methylation profiles of the *MLH1*, *MGMT*, and *DAPK-1* genes in cancerous tissues from different stomach sites.

**Methods:**

Samples were acquired from 81 patients suffering stomach adenocarcinoma who underwent surgery for gastric cancer in the Lithuanian University of Health Sciences Hospital Kaunas Clinics in 2009–2012. Gene methylation was investigated with methylation-specific PCR. The study was approved by the Lithuanian Biomedical Research Ethics Committee.

**Results:**

The frequencies of methylation in cancerous tissues from the upper, middle, and lower thirds of the stomach were 11.1, 23.1, and 45.4 %, respectively, for *MLH1*; 22.2, 30.8, and 57.6 %, respectively, for *MGMT*; and 44.4, 48.7, and 51.5 %, respectively, for *DAPK-1. MLH1* and *MGMT* methylation was observed more often in the lower third of the stomach than in the upper third (*p* < 0.05)*.* In the middle third, *DAPK-1* promoter methylation was related to more-advanced disease in the lymph nodes (N2–3 compared with N0–1 [*p* = 0.02]) and advanced tumor stage (stage III rather than stages I–II [*p* = 0.05]). *MLH1* and *MGMT* methylation correlated inversely when the tumor was located in the lower third of the stomach (coefficient, –0.48; *p* = 0.01). *DAPK-1* and *MLH1* methylation correlated inversely in tumors in the middle-third of the stomach (coefficient, –0.41; *p* = 0.01).

**Conclusion:**

Gene promoter methylation depends on the gastric tumor location.

## Background

Gastric cancer is a serious health problem. The concealed clinical progress of the illness means that this disease is typically diagnosed in its late stages, leading to poor prognoses, despite the new treatment opportunities that have emerged recently.

More than 90 % of stomach cancers are diagnosed as adenocarcinoma [1]. Since 1965, gastric adenocarcinoma has been divided into two main types, diffuse and intestinal (according to Lauren [2]). These two histological types differ in their etiologies and prognoses. Intestinal and stomach adenocarcinomas are often a consequence of chronic infection with *Helicobacter pylori*, when the normal gastric mucosa transforms to acute late chronic gastritis, intestinal metaplasia, and dysplasia [3]. Diffuse adenocarcinoma is more aggressive than the intestinal type, because its formation does not depend on gastritis or metaplasia processes [4].

Recent scientific data show that the prognosis of gastric adenocarcinoma depends not only on its histological type, but also on the tumor location in the stomach. Proximal tumors (originating in the cardia), which usually form in response to gastroesophageal acid/bile reflux [5], are now separated as a distinct and more aggressive form of gastric adenocarcinoma, which differ from distal stomach tumors.

Should distal stomach cancer be classified as unique? Cohort studies have shown that the risk of the premalignant condition, called ‘atrophic gastritis’, developing into cancer differs according to the site of the lesion in the stomach. The relative risk of gastric cancer occurring in patients with corpus-predominant gastritis is higher than in those with predominantly antral gastritis [6]. Does this fact indicate different carcinogenic processes in the stomach antrum and corpus? There are no data to either exclude or confirm this.

Epigenetic modifications play a central role in gastric carcinogenesis [7]. These processes, such as the methylation of CpG islands in gene promoter regions, cause reversible structural changes in the DNA, which in turn modify gene expression [8]. DNA methylation occurs when a methyl group (–CH_3_) is transferred to the 5^th^ position of cytosine [9].

A previous pilot study by our research group showed that the frequency of methylation of the *MLH1* promoter differs in the corpus and antral stomach tissues affected by pangastritis. We noticed that this epigenetic process is significantly more frequent in the antral gastritis tissues than in the corpus [10], whereas the degree of atrophic gastritis is similar in both regions. It is unclear whether this tendency is also true of cancerous tissues.

Two groups of genes were selected for this methylation analysis. The first group (*MLH1* and *MGMT*) contains two genes that encode proteins involved in repair of mutations and other DNA lesions before further cell division, and the second group contains a tumor-suppressing gene (*DAPK-1*) encoding a protein that induces apoptosis in the presence of a severe genomic defect.

The *MLH1* (mutL homolog 1, colon cancer, nonpolyposis type 2 (*E. coli*)) gene occurs at the 3p21.3 locus. The gene-encoded protein identifies DNA errors that appear after replication. Hypermethylation of the *MLH1* promoter, and not mutation-caused changes in the protein function, is responsible for the microsatellite instability in gastric cancer [11]. According to the literature, *MLH1* methylation is responsible for the intestinal form of gastric cancer and the early stage of the disease [12–16].

The function of MGMT (O^6^-methylguanine-DNA-methyltransferase) is to remove the alkyl group from the O^6^ position of guanine [17]. Disruption of the MGMT function may cause the emergence of mutations. Scientific publications indicate that the methylation of the *MGMT* gene promoter is one of the pathogenetic pathways in gastric cancer. In particular, gene methylation is attributed to the advanced stage of the disease [18], and is also attributed to the poorer disease prognosis [19].

The *DAPK-1* (death-associated protein kinase 1) gene is one of three *DAPK* family genes encoding calcium/calmodulin-dependent serine–threonine protein kinases. The function of the DAPK-1 protein is closely related to various apoptosis pathways, and *DAPK-1* hypermethylation has been shown to occur in solid and hematological tumors.

Gastric cancer is not an exception. Gene promoter methylation is also typical of adenocarcinomas in this location. In an earlier study, our group found an inverse association between *DAPK-1* and *MLH1* methylation [20]. We test here the hypothesis that different gene methylation profiles occur in cancerous tissues in the stomach according to the site of the tumor (upper, middle, or lower third).

## Methods

The study was approved by the Lithuanian Regional Biomedical Research Ethics Committee. All patients signed an informed consent form. Patients treated at the Lithuanian University of Health Sciences Hospital (LUHSH) Kaunas Clinics in 2009–2012 participated in the study. Stomach tissue samples were taken from all subjects with morphologically confirmed stage I–III adenocarcinoma. The study population consisted of 81 patients (34 men and 47 women) with a median age of 68 years (range 23–87 years; standard deviation [SD] 11.75). The tumors of nine patients were in the upper third of the stomach, those of 39 patients were in the middle third, and those of 33 patients were in the lower third of the stomach. The stomach tissue samples were collected from the tumor sites during surgery. These materials were immediately frozen in liquid nitrogen and stored at −80 °C until analysis. During the study, the patients’ clinical data (age at diagnosis, sex) and morphological data (tumor invasion according to the TNM classification, histological type according to the Lauren classification, and degree of differentiation according to the World Health Organization classification) were obtained from medical records.

### Methylation-specific polymerase chain reaction (MSP)

DNA was extracted from 25–30 mg of frozen tissue using the ZS Genomic DNA™ Tissue Mini Prep Kit (Zymo Research, USA), according to the manufacturer’s instructions. The methylation status of the *MLH1*, *MGMT*, and *DAPK-1* promoters was determined by treating the DNA with bisulfite, using the EZ DNA Methylation Gold Kit™ (Zymo Research), according to the manufacturer’s instructions. Human genomic DNA from peripheral blood lymphocytes, treated with bisulfite, was used as the negative control. Human genomic DNA treated *in vitro* with SssI methyltransferase (New England Biolabs, UK) was used as the positive control. The methylation status of the promoters was detected with MSP.

**Fig. 1:**
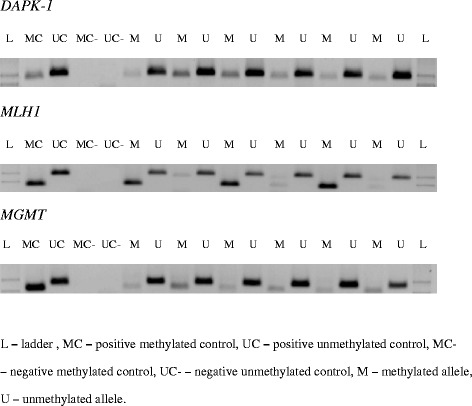
Representative samples of MSP analyses of DNA samples taken from stomach cancer tissues

The primers for the methylated and unmethylated DNA sequences are listed in Table 1. PCR was performed in a total reaction volume of 20 μL, containing 10 μL of Maxima® Hot Start PCR Master Mix with Hot Start *Taq* DNA Polymerase (Thermo Fisher Scientific, USA) and 10 μM of each primer (Metabion International AG, Germany). MSP was performed for 38–40 cycles of denaturation at 94 °C for 30 s, annealing for 1 min at the temperature appropriate for the individual gene, and extension at 72 °C for 1 min. The PCR products were separated by 3.5 % agarose gel electrophoresis (Fig. 1). When both methylated and unmethylated signals appeared on a gel, the gene was considered to be methylated.

**Table 1:** Primers used for MSP

Primer name	Forward sequence	Reverse sequence	Reference number
*MLH1* methylated	TATATCGTTCGTAGTATTC [GenBank:NG_0071092]	TCCGACCCGAATAAACC	[40]
*MLH1* unmethylated	TTTTGATGTAGATGTTTTATTAGGGT [GenBank:NG_0071092]	TACCACCTCATCATAACTACC	[40]
*MGMT* methylated	TTTCGACGTTCGTAGGTTTTCGC [GenBank:NG_000010.11]	GCACTCTTCCGAAAACGAAAC G	[40]
*MGMT* unmethylated	TTTGTGTTTTGATGTTTGTAGGTTTTTGT [GenBank:NG_000010.11]	AACTCCACACTCTTCCAAAAACAAAACA	[40]
*DAPK-1* methylated	GGATAGTCGGATCGAGTTAACGTC [GenBank:NG_029883.1]	CCCTCCCAAACGCCG A	[40]
*DAPK-1* unmethylated	GGAGGATAGTTGGATTGAGTTAATGTT [GenBank:NG_029883.1]	CAAATCCCTCCCAAACACCAA	[40]

*bp* base pairs

### Mathematical–statistical data analysis

Statistical data analysis was performed with the SPSS Statistics 19 software package (IBM SPSS Inc., USA). Quantitative properties were described by the arithmetic mean and standard deviation. Student’s *t*-test was used to verify the hypothesis of equal means. The *χ*^2^ criterion and Fisher’s exact test were used to check the statistical hypothesis of independence of two variables. Correlations between variables were evaluated by calculating Spearman’s coefficient. *p* < 0.05 was deemed significant.

## Results

The frequencies of promoter methylation in cancerous tissues from the upper, middle, and lower thirds of the stomach were 11.1, 23.1, and 45.4 %, respectively, for the *MLH1* gene; 22.2, 30.8, and 57.6 %, respectively, for the *MGMT* gene; and 44.4, 48.7, and 51.5 %, respectively, for the *DAPK-1* gene. Methylation of the *MLH1* and *MGMT* genes was observed more often in the lower third of the stomach than in the upper third (*p* < 0.05)*.* The detailed data are given in Table 2. A sex-based subgroup analysis revealed that *MLH1* methylation in the cancer tissues of the lower-third stomach was typical of the female population (*p* = 0.01), whereas *MGMT* methylation was only statistically significant in the male group (*p* = 0.03). The reasons for these findings are unclear, and require detailed analysis and further studies. The promoter methylation of the analyzed genes was not related to patient age.

**Table 2:** Distribution of *MLH1, MGMT*, and *DAPK-1* methylation in cancerous gastric tissues according to stomach site

	Stomach upper third cancerous tissue (9 patients) M/U	Stomach middle-third cancerous tissue (39 patients) M/U	Stomach lower-third cancerous tissue (33 patients) M/U	*p* value
*MLH1*	1/8	9/30	15/18	0.05
*MGMT*	2/7	12/27	19/14	0.01
*DAPK-1*	4/5	19/20	17/16	0.93

*M* methylated allele, *U* unmethylated allele

The two main histological classification categories – intestinal and diffuse types of tumors – reflect the differentiation of the tumor and the tendency of the cancerous cells to proliferate and spread. Therefore, we evaluated the possible associations between the frequency distributions of *DAPK-1*, *MLH1*, and *MGMT* methylation and the Lauren morphological tumor classification. The results are presented in Table 3. Because only a small number of patients had histologically confirmed mixed-type adenocarcinoma according to the Lauren classification (*n* = 7), this group was assigned to the more aggressive diffuse form of the disease.

**Table 3:** Distribution of *MLH1, MGMT, DAPK-1* methylation according to tumor site and pathological characteristics

	*MLH1 M/U*			*MGMT M/U*			*DAPK-1 M/U*		
Tumor histological parameters	Stomach upper-third cancerous tissue	Stomach middle-third cancerous tissue	Stomach lower-third cancerous tissue	Stomach upper-third cancerous tissue	Stomach middle-third cancerous tissue	Stomach lower-third cancerous tissue	Stomach upper-third cancerous tissue	Stomach middle-third cancerous tissue	Stomach lower-third cancerous tissue
Intestinal type	0/3	6/16	7/10	1/2	5/17	8/11	3/0	12/10	10/7
Diffuse type	1/5	3/14	8/8	1/5	7/10	11/5	1/5	7/10	7/9
*p* value	0.45	0.48	0.61	0.57	0.22	0.21	0.02	0.41	0.39
T1	0/1	1/2	2/4	0/1	1/2	4/2	1/0	2/1	4/2
T2	0/2	2/2	2/3	0/2	1/3	3/2	1/1	0/4	3/2
T3	0/2	5/11	8/9	1/1	4/12	10/7	1/1	6/10	7/10
T4	1/3	1/15	3/2	1/3	6/10	2/3	1/3	11/5	3/2
*p* value	0.7	0.17	0.83	0.62	0.88	0.84	0.59	0.06	0.67
N0	0/4	5/9	6/4	2/2	4/10	5/5	3/1	4/10	6/4
N1	0/2	2/4	3/5	0/2	2/4	5/3	0/2	2/4	4/4
N2	0/2	1/6	3/2	0/2	1/6	3/2	1/1	2/5	1/4
N3	1/0	1/11	3/7	0/1	5/7	6/4	0/1	11/1	6/4
*p* value	0.29	0.33	0.48	0.36	0.66	0.95	0.27	0.01	0.46

*M* methylated allele, *U* unmethylated allele

According to our data, methylation of the *DAPK-1* promoter was more frequent in intestinal-type gastric adenocarcinoma. However, this was merely a trend because the results were not statistically significant (*p* = 0.06). A subgroup analysis according to the tumor location revealed that the frequency of *DAPK-1* promoter methylation was significantly related to the intestinal cancerous tissue when the tumor was detected in the upper third of the stomach (*p* = 0.02; Table 3). No association was identified between the methylation of *MLH1* or *MGMT* and the tumor type according to the Lauren classification.

We also analyzed the association between the methylation frequency of the same genes and tumor invasion into the stomach wall (category T). The methylation of genes *MLH1* and *MGMT* was not associated with the T category of the TNM classification of the cancerous tissue in any part of the stomach. More frequent methylation of *DAPK-1* was observed in advanced tumors in the middle third of the stomach, but this was not statistically significant (*p* = 0.06), although this trend should be explored in the future.

The correlation between gene promoter methylation and lymph-node involvement was analyzed, but revealed no significant association between the methylation of the *MLH1* and *MGMT* promoters and the tumor lymph-node status. However, the apoptosis-related gene *DAPK-1* was more often methylated in the cancerous tissues in the N3 category of the TNM classification (when tumor metastasis is found in ≥ 7 regional lymph nodes) than in those in the N0–2 category (*p* = 0.05). A subgroup analysis showed that this association was only significant in the cancerous tissues of the middle third of the stomach (*p* = 0.01).

When the tumors were classified according to the TNM system, we found no association between the methylation of these genes and the tumor stage. However, a more precise analysis of the subgroups showed that the methylation of the *MLH1* promoter tended to be more frequent in cancerous tissues in the middle third of the stomach in stage I–II tumors than in stage III tumors (*p* = 0.06). Epigenetic changes in the *DAPK* gene were detected significantly more frequently in gastric cancerous tissue from the middle third of the stomach in stage III tumors than in stage I–II tumors (*p* = 0.05).

The results of our study indicate an inverse correlation between the methylation of the *MLH1* and *MGMT* genes in gastric cancer tissues when the tumor was located in the lower third of the stomach (coefficient, –0.48; *p* = 0.01). An inverse correlation between the methylation of *DAPK* and *MLH1* was also demonstrated in the cancerous tissue in the middle third of the stomach (coefficient, –0.41; *p* = 0.01).

## Discussion

### *MLH1*

According to the literature, the frequency of *MLH1* methylation in cancerous gastric tissues ranges from 14 to 43 % [12–16]. These wide variations in the frequency of *MLH1* methylation can be attributed to the different geographic regions considered in particular studies. The anatomical distribution of stomach tumor sites is known to differ in different geographic regions. In regions where the incidence of *H. pylori* infection is high (Eastern Europe, Eastern Asia), noncardial or distal gastric cancer is diagnosed most often. However, in Western and Northern Europe, where the number of *H. pylori-*infected people is quite low, the number of proximal gastric cancers is high. The scientific literature reports that the methylation of the *MLH1* promoter in stomach cancer tissues is significantly related to the *H. pylori* virulence factor encoded by the *vacs1* gene [21]. This gene, which is typically detected in the bacterial genome, provokes a more-intensive inflammatory response and is related to an increased risk of gastric cancer in patients with atrophic gastritis [22]. Chronic gastric mucosal inflammation, which is characterized by the mucosal infiltration of polymorphonuclear leukocytes, macrophages, and T and B lymphocytes, leads to the release of reactive oxygen species (ROS) from activated inflammatory cells. ROS induce DNA damage and the lack of a proper function of the mismatch repair system [23]. This is a multistep process that depends on the intensity of inflammation. The methylation of the *MLH1* promoter occurs in a late stage of atrophic gastritis, intestinal metaplasia [10, 24].

This consistent pattern is also supported by data from a study of patients from Western and Northern Europe [13]. Almost one third of the patients were diagnosed with proximal (cardial) gastric cancer, and the frequency of *MLH1* methylation was 14.2 %. This epigenetic change is also more frequently detected in distal gastric cancerous tissue [13]. The results of our study show that the occurrence of *MLH1* methylation in gastric cancer tissue increases from the upper and middle third of the stomach to the lower third, and was 11.1, 23.1, and 45.4 %, respectively. As in the literature, we also showed that this epigenetic change occurs significantly more often in the lower third of the stomach than in its proximal third, and this effect is more pronounced in the female population. The reason for sex related differences is unclear, further investigations are needed.

Some data have indicated that the methylation of the *MLH1* promoter is detected significantly more frequently in intestinal-type cancerous stomach tissues (according to the Lauren classification) than in the diffuse type [15, 16]. Our results show that the distribution of this epigenetic change is similar across tumor types according to Lauren classification.

We found no significant association between the methylation of *MLH1* and the T and N criteria for cancerous tissues in any part of the stomach. A separate analysis according to the tumor site showed that the epigenetic change (*MLH1* methylation) tended to be more frequent in cancerous tissue from the middle third of the stomach when in stage I–II tumors than in stage III tumors (*p* = 0.06). This result may not have been statistically significant because the number of patients with early-stage disease was relatively small.

We have found no published analysis of the relationship between this epigenetic change and the T, N, and stage criteria according to the tumor site in the stomach. However, published data indicate that, in general, the methylation of *MLH1* in cancerous tissues is detected more often when regional lymph nodes are not involved [14].

### *MGMT*

The rate of methylation of the *MGMT* promoter in cancerous tissues increased from the upper to the lower third of the stomach (22.2, 30.8, and 57.6 %, respectively), and this effect is more pronounced in the male population. The reason for sex related differences is unclear, further investigations are needed. According to the literature, *MGMT* methylation in cancerous stomach tissues ranges from 7 to 61 % [13, 25–28]. This wide variation could be explained by the fact that this epigenetic change is detected more often in distal stomach cancer tissue than at proximal sites [18, 29].

We found no significant associations between *MGMT* promoter methylation and the T and N criteria or the histological type of the tumor according to the Lauren classification at any investigated stomach site. The loss of the MGMT protein function is related to tumor spread to the lymph nodes [14, 18] and advanced stages of the disease [18, 30]. In our study, the number of patients with early-stage disease was comparatively small, which could explain our findings.

### *DAPK-1*

In this study, the frequency of *DAPK-1* promoter methylation did not correlate with tumor location. Published data show that its frequency varies from 22 to 91 % [28, 31–36]. According to our data, methylation of the *DAPK-1* promoter was more frequent in the intestinal type of gastric adenocarcinoma. However, this was merely a trend because the differences were not significant (*p* = 0.06). A subgroup analysis according to the tumor location showed that the frequency of *DAPK* promoter methylation was significantly higher in intestinal cancerous tissues in the upper third of the stomach. We found no associations mentioned in literature between *DAPK-1* promoter methylation and tumor classification according to the Lauren. Our results should be interpreted critically because of the few patients in this subgroup.

We noted a marginal trend in which *DAPK-1* methylation was detected more frequently in the middle third of the stomach in more advanced tumors, according to the T criterion (*p* = 0.06). We also found that when the apoptosis-related gene *DAPK-1* was methylated in cancerous tissues, the tumor was more likely to be N3 on the TNM classification (tumor metastases found in ≥ 7 regional lymph nodes) than N0–2 (*p* = 0.05) and to be an advanced tumor (stage III rather than stages I–II; *p* = 0.05). A subgroup analysis showed that this association occurs in cancerous tissues from the middle third of the stomach (p-0.01), but is not significant at other stomach sites. A study published in 2010 showed that the methylation of the pro-apoptotic gene *DAPK-1* in cancerous stomach tissues is related to tumor spread to the lymph nodes [37], but we did not find any information in the mentioned study linking these findings to different sites of stomach cancer localizations.

### Correlation between the methylation status of the analyzed genes

The relationships between the promoter methylation of the different genes in stomach cancer tissues were examined our study. Do these correlations depend on the tumor site? We have found no information in the scientific literature regarding the correlation of *MGMT* and *MLH1* promoter methylation in stomach adenocarcinoma tissues. The results of our study indicate that there was an inverse correlation between the methylation of *MLH1* and *MGMT* in cancer tissues when the tumor was located in the lower third of the stomach (coefficient, –0.48; *p* = 0.01).

These results are not surprising because the methylation of both gene promoters is more often detected in the lower-stomach cancer tissues [13, 29]. However, the proteins encoded act in different stages of carcinogenesis. *MLH1* promoter methylation is more often detected in the early stages and in less aggressive gastric tumors [12–16], whereas *MGMT* promoter methylation is more typical of late-stage stomach adenocarcinomas and poor prognoses [18, 19].

More information is available about the association between the promoter methylation of *MLH1* and *DAPK-1*. High microsatellite instability correlates inversely with the methylation of the *DAPK-1* promoter [12]. Our research group identified an inverse, albeit weak, correlation between the promoter methylation of *DAPK-1* and *MLH1*, regulating microsatellite function, in cancerous stomach tissues [20].

We also found that this correlation occurs in the middle third of the stomach. An inverse correlation between the methylation of *DAPK-1* and *MLH1* was also identified in cancer tissues sampled from the middle third of stomach (coefficient, –0.41; *p* = 0.01).

The specific limitation of this study is that no information on the mRNA expression of the analyzed genes was provided. Our team plans to undertake this with quantitative PCR in a future study. The current findings are important not only because they extend our understanding of gastric carcinogenesis, but also from the clinical perspective. There is a growing body of evidence that gene methylation in cancerous gastric tissues is associated with differential sensitivity to chemotherapy. Methylation of the *MLH1* gene is related to resistance to oxaliplatin-based chemotherapy [38], and patients with *DAPK-1* methylation show a worse response to fluoropyrimidine-based chemotherapy [39]*.*

We hope that our study will lead to better understanding of the different pathways of molecular carcinogenesis in different parts of the stomach.

## Conclusion

Our study results show that an epigenetic process, the methylation of gene promoters, in cancerous tissue depends on the location of the gastric tumor.

## Abbreviations

bp: base pairs
*DAPK-1*: death-associated protein kinase 1
LUHSH: Lithuanian University of Health Sciences Hospital
M: methylated allele
*MGMT*: O^6^-methylguanine-DNA-methyltransferase
MC: positive methylated control
MC-: negative methylated control
*MLH1*: mutL homolog 1, colon cancer, nonpolyposis type 2 (*E. coli*)
mRNA: messenger RNA
MSP: methylation-specific polymerase chain reaction
PCR: polymerase chain reaction
ROS: reactive oxygen species
U: unmethylated allele
UC: positive unmethylated control
UC-: negative unmethylated control
